# Retraction: Assessing the Knowledge and Perceptions of Medical Students After Using Kahoot! in Pharmacology Practical Sessions at King Abdulaziz University, Jeddah

**DOI:** 10.7759/cureus.r214

**Published:** 2026-01-02

**Authors:** Lana Shawwa, Fatemah Kamel

**Affiliations:** 1 Medical Education, King Abdulaziz University, Jeddah, SAU; 2 Pharmacology Department, King Abdulaziz University Faculty of Medicine, Jeddah, SAU

This article has been retracted by the Editor-in-Chief due to flawed study design and improper statistical analyses. The same students were analyzed as if they were separate groups, and many statistical tests were run without proper correction, increasing the chance of false positive findings. Score increases are also likely influenced by students repeating the same test questions and by questionable use of exam difficulty measures. Overall, the results are more likely explained by topic differences and testing effects than by a real educational benefit from Kahoot!. The authors disagree with the decision to retract.

